# Baseball Player Behavior Classification System Using Long Short-Term Memory with Multimodal Features

**DOI:** 10.3390/s19061425

**Published:** 2019-03-22

**Authors:** Shih-Wei Sun, Ting-Chen Mou, Chih-Chieh Fang, Pao-Chi Chang, Kai-Lung Hua, Huang-Chia Shih

**Affiliations:** 1 Department of New Media Art, Taipei National University of the Arts, Taipei 112, Taiwan; 2 Computer Center, Taipei National University of the Arts, Taipei 112, Taiwan; 3 Department of Communication Engineering, National Central University, Taoyuan 320, Taiwan; tcmou.vaplab@gmail.com (T.-C.M.); pcchang@ce.ncu.edu.tw (P.-C.C.); 4 Graduate Institute of Dance Theory, Taipei National University of the Arts, Taipei 112, Taiwan; m10446017@dance.tnua.edu.tw; 5 Department of Computer Science and Information Engineering, National Taiwan University of Science and Technology, Taipei 106, Taiwan; hua@mail.ntust.edu.tw; 6 Center for Cyber-Physical System Innovation, National Taiwan University of Science and Technology, Taipei 106, Taiwan; 7 Department of Electrical Engineering, Yuan Ze University, Taoyuan 320, Taiwan; hcshih@saturn.yzu.edu.tw

**Keywords:** behavior recognition, multimodal, machine learning, deep learning, LSTM network, depth camera, inertial sensor

## Abstract

In this paper, a preliminary baseball player behavior classification system is proposed. By using multiple IoT sensors and cameras, the proposed method accurately recognizes many of baseball players’ behaviors by analyzing signals from heterogeneous sensors. The contribution of this paper is threefold: (i) signals from a depth camera and from multiple inertial sensors are obtained and segmented, (ii) the time-variant skeleton vector projection from the depth camera and the statistical features extracted from the inertial sensors are used as features, and (iii) a deep learning-based scheme is proposed for training behavior classifiers. The experimental results demonstrate that the proposed deep learning behavior system achieves an accuracy of greater than 95% compared to the proposed dataset.

## 1. Introduction

Human action recognition is widely applied in many smart environment applications. For example, video surveillance, interactive gaming, and health monitoring need to recognize different actions performed by a human subject. Triggered by the recognized actions and behaviors, a smart system can respond the possible alert to a police department, interactive audio and visual effects, and medical assisting. To achieve human action recognition, camera sensors [1,2,3,4] are used to obtain the intensities from a visual sensor mounted in a camera. To overcome the high computational cost from processing RGB images on a camera for the 3D skeletal joint positions, depth cameras [5,6,7,8,9,10] with infra-red (IR) emitters and receiving sensors are used to obtain the 3D data from skeletal joints. Inertial sensors, such as accelerometers and gyro sensors [11,12,13], mounted in wearable devices are used to measure the movement of a user for hand gesture recognition.

**Figure 1:**
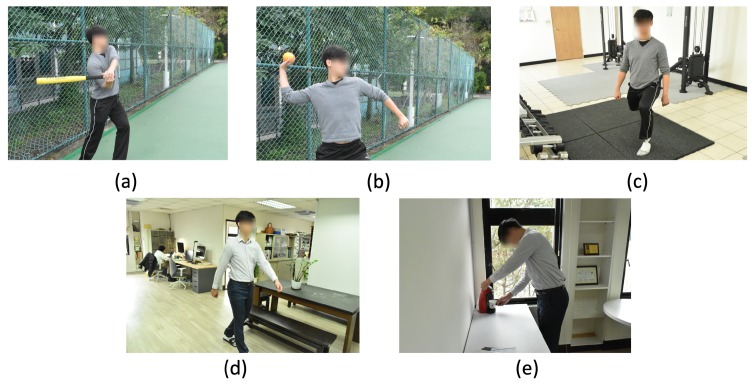
The proposed baseball player behavior classification system: (**a**) the swing behavior of a baseball player, (**b**) the pitch behavior of a baseball player, (**c**) a warm-up exercise, (**d**) the normal walking of a baseball player outside the sport, and (**e**) coffee-pouring behavior in daily life, outside the sport.

To train a potential baseball player, on- and off-field behaviors have equal value for performance evaluation. Most research has addressed only on-field activities. However, both on- and off-field activities are essential for comprehending player performance and level. When a baseball coach chooses which player should be included in a game, not all of players’ off-field behaviors are known to him or her. Therefore, in this paper, we propose a baseball player behavior classification system to provided coaches with player performance evaluation. In the on-field situations, the swinging and pitching behaviors recognized by the proposed system are recognized (see Figure 1a,b). In the off-field situations, before a game, the proposed system tracks a baseball player’s warm up behaviors, level, and times. In daily life, a potential baseball player should keep in a proper walking manner, and the proper walking behaviors can be recognized and displayed on a smart glass. Moreover, to prevent caffeine overdosing that might mislead a baseball player’s evaluation results, coffee-pouring (CP) behaviors can be recognized, quantified, and reported by the proposed system.

Therefore, in this paper, we propose a behavior recognition scheme combining information captured from a depth camera sensor and inertial sensors worn on a human subject. To achieve behavior recognition, the contribution of this paper is threefold: (i) segment and fuse a user’s sensory data from a depth camera and multiple inertial sensors, (ii) extract features from the reliable skeletal position based on time-variant skeleton vector projection from a depth camera and on statistical properties from the inertial sensors, and (iii) train behavior classifiers based on a deep-learning approach with decision-level fusion. The rest of this paper is organized as follows. Relevant research is discussed in Section 2, The proposed behavior recognition system is described in detail in Section 3. Experimental results are evaluated in Section 4. Finally, the conclusions are given in Section 5.

## 2. Related Work

For behavior recognition, Zelnik-Manor and Irani [1] proposed a stationary video camera analyzing spatiotemporal features with a statistical distance measure for action recognition. In addition, with Hidden Markov Model (HMM) training, Khan and Sohn [2] proposed analyzing video frames obtained from two cameras capturing two viewpoints for abnormal human activity recognition. In addition, to recognize human movements from a large video database, Kuehne et al. [3] proposed manually annotating around 7000 video clips for motion recognition. Moreover, Mehta et al. [4] proposed using a single RGB camera for 3D evaluation of human poses. However, from a monochromatic camera, obtaining real 3D information in a real space for human behavior recognition is challenging.

To measure precise 3D information in a real space, infrared-based depth cameras (Kinect) are widely used for behavior recognition. Shotten et al. [5] used the Microsoft Kinect camera to quickly and accurately recognize human poses from depth images. Luber et al. [6] used multiple depth cameras with online boosted target models to track moving human subjects. To more precisely obtain 3D information on a human subject, Zollhofer et al. [7] used RGBD information from the Kinect camera to reconstruct the 3D point cloud for nonrigid parts of a human subject. To obtain complex postures in challenging scenes, Dou et al. [8] used multiple depth cameras for performance capturing based on spatiotemporal coherent properties. To more precisely measure 3D information, Newcombe et al. [9] proposed using dense simultaneous localization and mapping processes to reconstruct nonrigid scenes from RGBD information for scanning moving objects and scenes. Orts-Escolano et al. [10] used the RGBD streams with foreground human subject segmentation for virtual 3D teleportation in AR and VR displays. However, although 3D information and the surface information can be more precisely measured from depth cameras, inertial movement of a user’s body parts cannot be obtained from depth cameras, only the environment for behavior recognition.

Inertial sensors may be helpful for behavior recognition. Xu et al. [11] used a mounted MEMS accelerometer for user hand-gesture recognition. Gupta et al. [12] used accelerometer and gyroscope sensors mounted in a smart device for hand gesture recognition. Furthermore, Xie and Cao [13] used neural networks to train hand gesture models from accelerometers. However, the mounted inertial sensors (accelerometers or gyro sensors) can recognize hand gestures, but human behaviors involving body movements remain a challenge.

Therefore, based on the aforementioned research, herein, we propose obtaining signals from a depth camera and wearable sensors to generate the fused classifiers for behavior recognition to be used in smart baseball applications.

## 3. Proposed Machine Learning Based Behavior Recognition Fusion System

**Figure 2:**
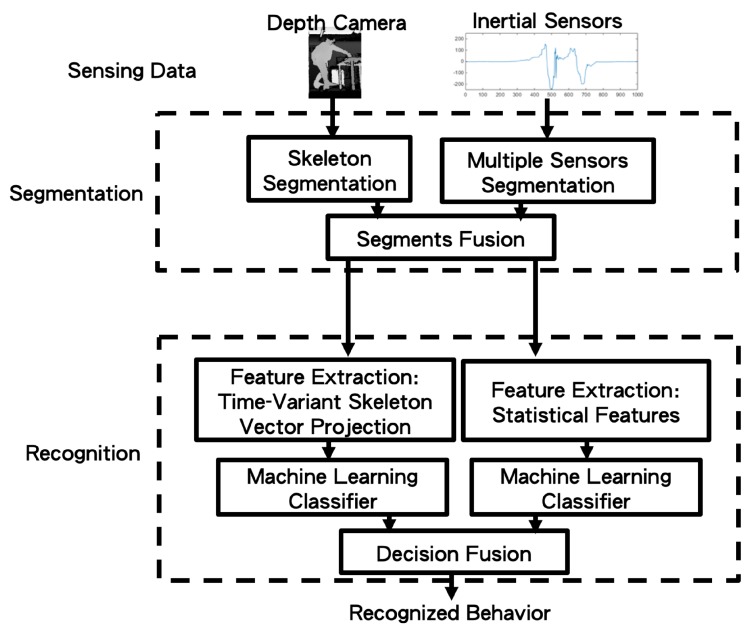
The system block diagram of the proposed method.

In this paper, as in Figure 2, user movements are captured by a depth camera and multiple wearable inertial sensors. After the segmentation process, the behaviors can be recognized by decision fusion. Detailed descriptions of the aforementioned processes follow.

### 3.1. Segmentation from Multimodal Sensors

**Figure 3:**
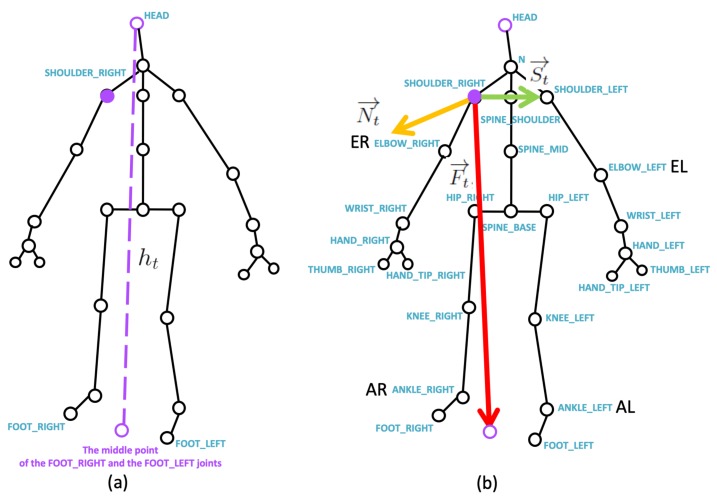
The joints according to Kinect SDK: (**a**) the normalization factor of the body height in each frame, and (**b**) the basis vectors for time-variant skeleton vector projection.

Kinect V2 [14] with the official Microsoft Kinect SDK 2.0 [15] are used to obtain the skeleton joints from the depth camera. Motivated by the segmentation process of Dawar and Kehtarnavaz’s method [16], for the skeleton joints (the empty circles) in Figure 3a, the 3D centroid position $\left(c_{x,t},c_{y,t},c_{z,t}\right)$ at time *t* is obtained as:

(1) $$\left(c_{x,t},c_{y,t},c_{z,t}\right)=\left(\frac{\sum_{i=1}^{N}x_{t,i}}{N},\frac{\sum_{i=1}^{N}y_{t,i}}{N},\frac{\sum_{i=1}^{N}z_{t,i}}{N}\right),$$

where the setting N=25 is used for Kinect V2. In addition, a centroid difference $C_{d,t}$ at the time *t* is calculated as:

(2) $$C_{d,t}=\sqrt{{\left(c_{x,t}-c_{x,t-1}\right)}^{2}+{\left(c_{y,t}-c_{y,t-1}\right)}^{2}+{\left(c_{z,t}-c_{z,t-1}\right)}^{2}}.$$

As suggested by the authors in Ref. [16], the frames with centroid differences above the 5% level of the maximum centroid difference to zero are treated as a movement or action.

**Figure 4:**
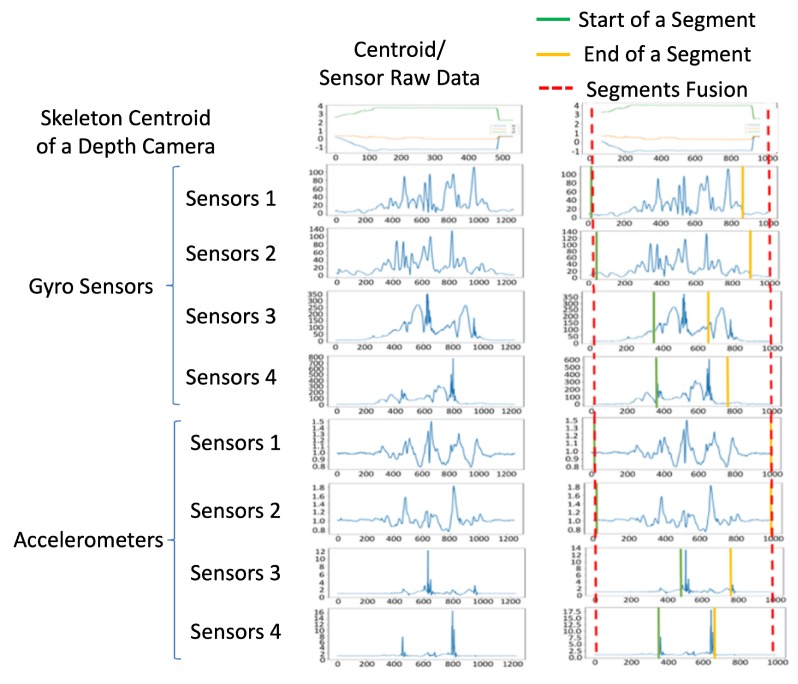
Segmentation fusion: raw data of the sensing signals from multiple sensors are on the left, and the resampled data are on the right.

For the inertial sensors, a similar segmentation process is operated on each sensor. Given the gyroscope sensor value $\left(g_{x,t,k},g_{y,t,k},g_{z,t,k}\right)$ and the accelerometer value $\left(a_{x,t,k},a_{y,t,k},a_{z,t,k}\right)$ at time *t* of the *k*-th sensor, the gyroscope difference $G_{d,t,k}$ and the accelerometer difference $A_{d,t,k}$ of the *k*-th sensor are calculated as follows:

(3) $$G_{d,t,k}=\sqrt{{\left(g_{x,t,k}-g_{x,t-1,k}\right)}^{2}+{\left(g_{y,t,k}-g_{y,t-1,k}\right)}^{2}+{\left(g_{z,t,k}-g_{z,t-1,k}\right)}^{2}},$$

and

(4) $$A_{d,t,k}=\sqrt{{\left(a_{x,t,k}-a_{x,t-1,k}\right)}^{2}+{\left(a_{y,t,k}-a_{y,t-1,k}\right)}^{2}+{\left(a_{z,t,k}-a_{z,t-1,k}\right)}^{2}},$$

respectively. Similarly, the gyroscope and accelerometer differences above the 5% level of the maximum gyroscope and accelerometer difference from zero are treated as the presence of movement or action for the *k*-th sensor. In the proposed method, k=1,⋯,4 for four sensors worn on a user’s body parts. An example for multiple sensing signals is shown in the left part of Figure 4.

The left part of Figure 4 features raw data obtained from different sampling rates in different sensor modalities, and the values from different sensors are resampled to the same number of points, as shown in the right of Figure 4. From top to bottom, the example of centroid movement from the depth sensor, and the sensor values of the gyro sensors and accelerometers are correspondingly displayed. Our observation is that the value changing from sensor modality is more sensitive than centroid movement in camera modality. Therefore, Equations (3) and (4) are used to detect the start point (green lines) and endpoint (yellow lines) in the right part of Figure 4, respectively. In the segment fusion process of Figure 2, by obtaining the combined set of the segments, the minimum values of the start points and the maximum values of the endpoints of the segments from multiple sensors, the behavior segments are bounded by the red dotted lines shown in the right part of Figure 4. Moreover, the corresponding data in the same time segment (signals bounded by the red dotted lines in the top right of Figure 4) obtained from the depth camera, gyro sensors, and accelerometers are used for further feature extraction.

### 3.2. Behavior Recognition

#### 3.2.1. Features from a Depth Camera: Time-Variant Skeleton Vector Projection

According to our previous research [17], given a 3D joint position $j_{t,i}=\left(x_{t,i},y_{t,i},z_{t,i}\right)$ (i=1,⋯,25 in Kinect V2), by measuring the distance $h_{t}$ from the 3D position of the head joint to the middle point between the FOOT RIGHT joint and the FOOT LEFT joint in Figure 3a, the normalized relative 3D joint position is as follows:

(5) $$j_{t,i}^{\prime }=\frac{\left(x_{t,i}-x_{t}^{sr},y_{t,i}-y_{t}^{sr},z_{t,i}-z_{t}^{sr}\right)}{h_{t}},$$

where $\left(x_{t}^{sr},y_{t}^{sr},z_{t}^{sr}\right)$ is the 3D position of the SHOULDER_RIGHT joint (purple dot in the center of Figure 3a) as the 3D origin point, and the prime symbol represents the normalized relative position. The normalization process by the body height $h_{t}$ has a user-invariant (users with different heights) property.

Taking the SHOULDER_RIGHT joint as a starting 3D starting point, a shoulder vector $\vec{S_{t}}$ from the SHOULDER_RIGHT joint to the SHOULDER_LEFT joint is depicted by the green arrow in Figure 3b. A foot vector $\vec{F_{t}}$ from the SHOULDER_RIGHT joint to the middle point (purple circle) between the FOOT RIGHT joint and the FOOT LEFT joint is depicted by the red arrow in Figure 3b. Based on the obtained shoulder vector $\vec{S_{t}}$ and the foot vector $\vec{F_{t}}$, a normal vector, $\vec{N_{t}}$ such that

(6) $$\vec{N_{t}}=\vec{S_{t}}\times \vec{F_{t}},$$

can be calculated using the cross product of $\vec{S_{t}}$ and $\vec{F_{t}}$, and $\vec{N_{t}}$ is depicted by the yellow arrow in Figure 3b. At time *t*, $\left\{\vec{N_{t}},\vec{S_{t}},\vec{F_{t}}\right\}$ are treated as the basis vectors.

Taking the relative normalized joint $j_{t,i}^{\prime }$ in a 3D space, the vector $\vec{j_{t,i}^{\prime }}$ can be used to obtain the projection amount to the basis vectors $\left\{\vec{N_{t}},\vec{S_{t}},\vec{F_{t}}\right\}$, and the feature $f_{t,i}$ is defined as:

(7) $$f_{t,i}=\left[f_{t,i}^{N}\;f_{t,i}^{F}\;f_{t,i}^{S}\right]=\left[\begin{array}{ccc} <\vec{N_{t}},\vec{j_{t,i}^{\prime }}> & <\vec{F_{t}},\vec{j_{t,i}^{\prime }}> & <\vec{S_{t}},\vec{j_{t,i}^{\prime }}> \end{array}\right..$$

Furthermore, because Kinect V2 is used here, 25 joints are obtained from the official SDK. Therefore, the feature $\phi_{t}$ at time *t* is expressed as:

(8) $$\phi_{t}=\left[\begin{array}{ccc} f_{t,i=1}^{N} & f_{t,i=1}^{F} & f_{t,i=1}^{S}\\ f_{t,i=2}^{N} & f_{t,i=2}^{F} & f_{t,i=2}^{S}\\ \vdots & \vdots & \vdots \\ f_{t,i=25}^{N} & f_{t,i=25}^{F} & f_{t,i=25}^{S} \end{array}\right],$$

**Figure 5:**
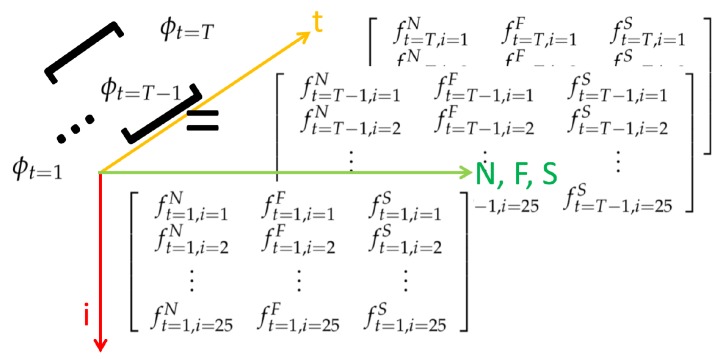
Feature space, camera modality.

Moreover, each feature $\phi_{t}$ at time *t* can be conceived of a slice (bottom part shown in Figure 5) of a spatio-temporal cube. By concatenating multiple slices along the temporal axis, a feature set $\phi_{T}$ can be expressed as:

(9) $$\phi_{T}=\left[\left[\phi_{t=1}\right],\cdots ,\left[\phi_{t=T-1}\right],\left[\phi_{t=T}\right]\right],$$

and the corresponding spatio-temporal representation is depicted in Figure 5.

#### 3.2.2. Features from the Inertial Sensors: Statistical Features

Given the mean $\mu_{g,k}$, standard deviation $\sigma_{g,k}$, and variance $\sigma_{g,k}^{2}$ of the the *k*-th gyroscope sensor in the *x*, *y* and *z* directions, and the mean $\mu_{a,k}$, standard deviation $\sigma_{a,k}$, and variance $\sigma_{a,k}^{2}$ of the the *k*-th accelerometer in the *x*, *y* and *z* directions, the features extracted from the gyro sensor $\eta_{w}$ and the accelerometer $\alpha_{w}$ are calculated as:

(10) $$\eta_{w}=\left[\begin{array}{c} \eta_{w,k=1}\\ \eta_{w,k=2}\\ \eta_{w,k=3}\\ \eta_{w,k=4} \end{array}\right]=\left[\begin{array}{ccccccccc} \mu_{g,x,k=1} & \mu_{g,y,k=1} & \mu_{g,z,k=1} & \sigma_{g,x,k=1} & \sigma_{g,y,k=1} & \sigma_{g,z,k=1} & \sigma_{g,x,k=1}^{2} & \sigma_{g,y,k=1}^{2} & \sigma_{g,z,k=1}^{2}\\ \mu_{g,x,k=2} & \mu_{g,y,k=2} & \mu_{g,z,k=2} & \sigma_{g,x,k=2} & \sigma_{g,y,k=2} & \sigma_{g,z,k=2} & \sigma_{g,x,k=2}^{2} & \sigma_{g,y,k=2}^{2} & \sigma_{g,z,k=2}^{2}\\ \mu_{g,x,k=3} & \mu_{g,y,k=3} & \mu_{g,z,k=3} & \sigma_{g,x,k=3} & \sigma_{g,y,k=3} & \sigma_{g,z,k=3} & \sigma_{g,x,k=3}^{2} & \sigma_{g,y,k=3}^{2} & \sigma_{g,z,k=3}^{2}\\ \mu_{g,x,k=4} & \mu_{g,y,k=4} & \mu_{g,z,k=4} & \sigma_{g,x,k=4} & \sigma_{g,y,k=4} & \sigma_{g,z,k=4} & \sigma_{g,x,k=4}^{2} & \sigma_{g,y,k=4}^{2} & \sigma_{g,z,k=4}^{2} \end{array}\right],$$

and

(11) $$\alpha_{w}=\left[\begin{array}{c} \alpha_{w,k=1}\\ \alpha_{w,k=2}\\ \alpha_{w,k=3}\\ \alpha_{w,k=4} \end{array}\right]=\left[\begin{array}{ccccccccc} \mu_{a,x,k=1} & \mu_{a,y,k=1} & \mu_{a,z,k=1} & \sigma_{a,x,k=1} & \sigma_{a,y,k=1} & \sigma_{a,z,k=1} & \sigma_{a,x,k=1}^{2} & \sigma_{a,y,k=1}^{2} & \sigma_{a,z,k=1}^{2}\\ \mu_{a,x,k=2} & \mu_{a,y,k=2} & \mu_{a,z,k=2} & \sigma_{a,x,k=2} & \sigma_{a,y,k=2} & \sigma_{a,z,k=2} & \sigma_{a,x,k=2}^{2} & \sigma_{a,y,k=2}^{2} & \sigma_{a,z,k=2}^{2}\\ \mu_{a,x,k=3} & \mu_{a,y,k=3} & \mu_{a,z,k=3} & \sigma_{a,x,k=3} & \sigma_{a,y,k=3} & \sigma_{a,z,k=3} & \sigma_{a,x,k=3}^{2} & \sigma_{a,y,k=3}^{2} & \sigma_{a,z,k=3}^{2}\\ \mu_{a,x,k=4} & \mu_{a,y,k=4} & \mu_{a,z,k=4} & \sigma_{a,x,k=4} & \sigma_{a,y,k=4} & \sigma_{a,z,k=4} & \sigma_{a,x,k=4}^{2} & \sigma_{a,y,k=4}^{2} & \sigma_{a,z,k=4}^{2} \end{array}\right],$$

where the statistical features are calculated in the time period from the time partitions of $\frac{T}{M}\cdot w$ for w=1,⋯,M. The parameter M=6 is adopted from the setting suggested in Ref. [18].

### 3.3. Machine Learning Classifiers with Decision Fusion

**Figure 6:**
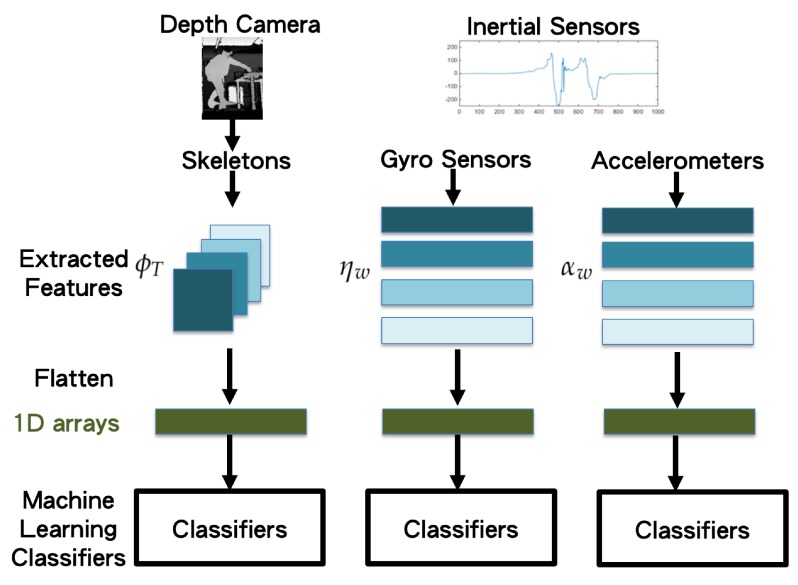
Flattened 1D vectors of the extracted feature vectors.

When the features $\phi_{t}$ in Equation (8), $\eta_{w}$ in Equation (10), and $\alpha_{w}$ in Equation (11) from the depth camera modality, gyro sensor modality, and accelerometer modality are obtained, before training the classifiers, the feature vectors from the three modalities are correspondingly flattened as 1D vectors:

(12) $$\Psi_{t}=\left[\begin{array}{cccccccccc} f_{t,i=1}^{N} & f_{t,i=1}^{F} & f_{t,i=1}^{S} & f_{t,i=2}^{N} & f_{t,i=2}^{F} & f_{t,i=2}^{S} & \cdots & f_{t,i=25}^{N} & f_{t,i=25}^{F} & f_{t,i=25}^{S} \end{array}\right],$$

(13) $$\eta_{w}=\left[\begin{array}{cccc} \eta_{w,k=1} & \eta_{w,k=2} & \eta_{w,k=3} & \eta_{w,k=4} \end{array}\right],$$

and

(14) $$\alpha_{w}=\left[\begin{array}{cccc} \alpha_{w,k=1} & \alpha_{w,k=2} & \alpha_{w,k=3} & \alpha_{w,k=4} \end{array}\right],$$

as seen in the processes depicted in Figure 6. Furthermore, by concatenating the 1D vectors obtained from different times *t* and observing window *w*, the concatenated 1D array from the three modalities are obtained as:

(15) $$\Psi =\left[\begin{array}{cccc} \Psi_{t=1} & \Psi_{t=2} & \cdots & \Psi_{t=T} \end{array}\right],$$

(16) $$\eta =\left[\begin{array}{cccccc} \eta_{w=1} & \eta_{w=2} & \eta_{w=3} & \eta_{w=4} & \eta_{w=5} & \eta_{w=6} \end{array}\right],$$

and

(17) $$\alpha =\left[\begin{array}{cccccc} \alpha_{w=1} & \alpha_{w=2} & \alpha_{w=3} & \alpha_{w=4} & \alpha_{w=5} & \alpha_{w=6} \end{array}\right].$$

**Figure 7:**
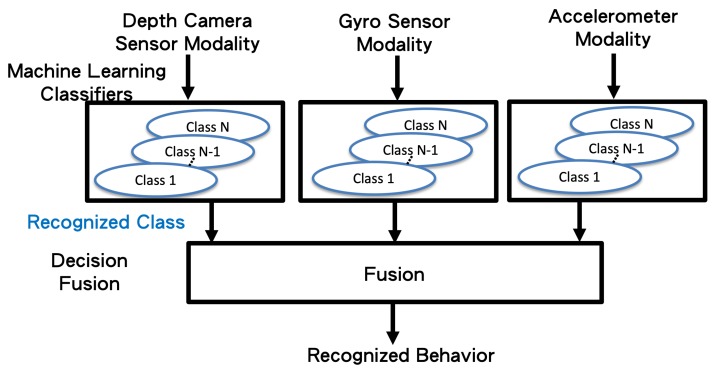
The decision fusion process of the proposed method.

**Figure 8:**
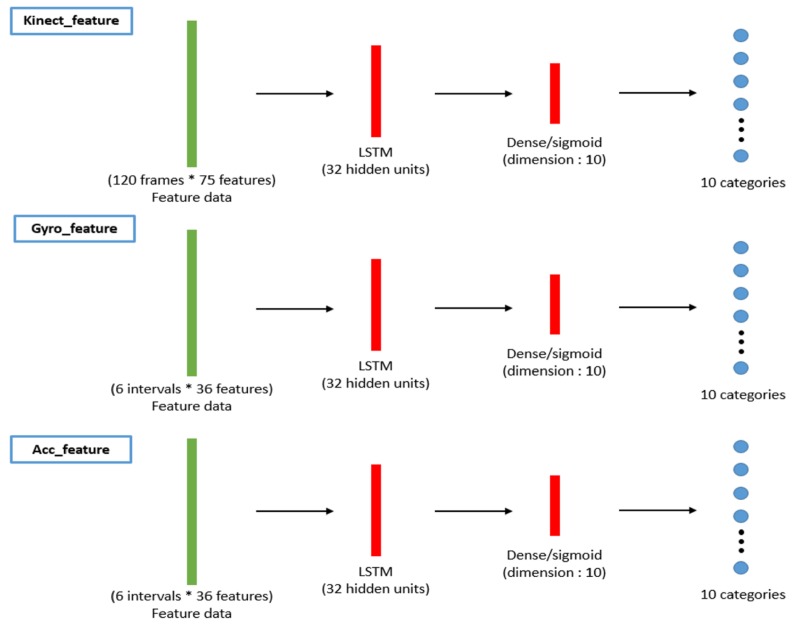
Training process for the behavior classifiers of the proposed LSTM-based scheme.

Concatenated one-dimensional features (camera, gyro sensors, and accelerometer) are taken as the input (bottom of Figure 6) of the machine learning classifiers (top of Figure 7). Herein, we propose applying the long short-term memory (LSTM) [19] network to use the sequential relationship for training the behavior classifiers among multiple modalities, as shown in Figure 8. According to the flattened 1D feature vectors obtained in Equations (12)–(17), from the depth camera sensor, gyro sensors, and accelerometers-sensing modalities, the corresponding 1D features (the green rectangles in Figure 8) are trained by the LSTM networks to obtain the behavior models (10 categories in the experiments in this paper), as shown from top to bottom in Figure 8. The hidden units for LSTM is set as 32 (the number of the hidden units of the proposed LSTM approach is evaluated from 8 to 256 with the power of 2. The accuracy performance is saturated after 32 hidden units. Moreover, the execution time always increases as the number of hidden units grows. Therefore, in this paper, the setting of 32 hidden units is chosen for implementing the proposed method), and the dimension of dense/sigmoid is set as 10 for classifying 10 categories. After the signals in each modality can be classified, a decision fusion (Level-1 fusion in Ref. [20]; Figure 7) from all modalities (herein, three modalities, namely depth sensor, gyro sensors, accelerometers) are operated through a majority voting process.

## 4. Experimental Results

**Figure 9:**
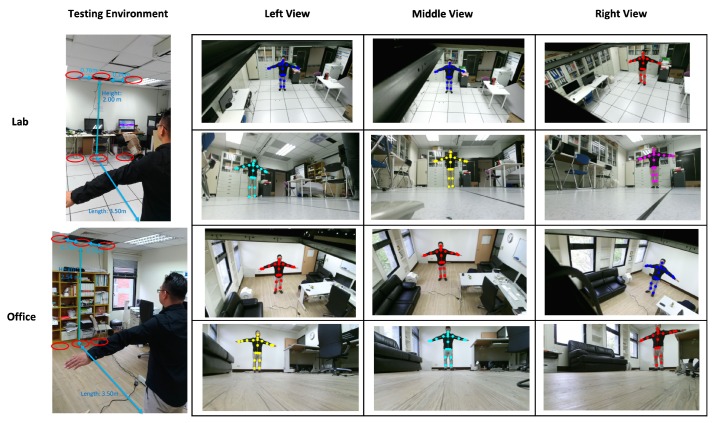
Skeletal joints obtained from Kinect SDK 2.0 [15] in “Lab” and “Office” environments.

**Figure 10:**
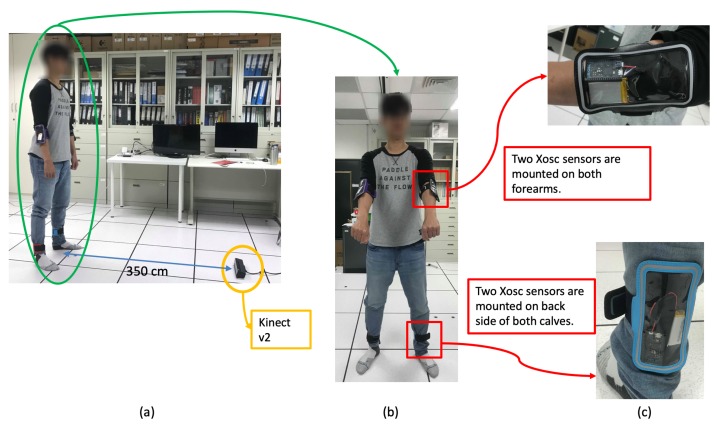
Testing environment: (**a**) the distance from the mounted depth sensor on the floor and the user, (**b**) the user wearing inertial sensors, and (**c**) the inertial sensors worn on the elbows and the ankles.

**Figure 11:**
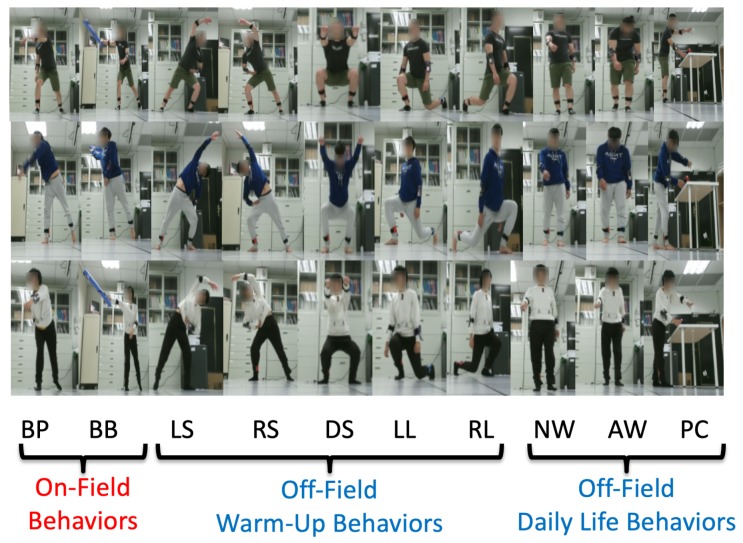
Ten on- and off-field behaviors performed by users: baseball pitch (BP), baseball bat (BB), left stretch (LS), right stretch (RS), deep squat (DS), left lunge (LL), right lunge (RL), normal walking (NW), abnormal walking (AW), and coffee pouring (CP).

In the experimental results, a Kinect V2 [14] depth camera is used to obtain the depth sensing information, and the skeletons of a user are obtained from the official Microsoft Kinect SDK 2.0 [15]. The skeletal joint obtained from the depth camera mounted in `Lab’ and `Office’ results are shown in Figure 9, and it demonstrates that the camera can be mounted in many side view places for daily use. In the experimental results, the depth sensor, the Kinect V2 depth camera, is mounted on the floor in front of a user, and the distance from the camera to the user is about 3.5 m, as shown in Figure 10a. The obtained body height $h_{t}$ in Equation (5) from all behavior tests are in the range between 1.29 m and 1.80 m. In addition, the inertial sensors, the x-OSC [21] sensors with a built-in Wi-Fi communication capability, are worn on the elbows (EL and ER in Figure 3b) and the ankles (AL and AR in Figure 3b) of the user, as shown in Figure 10b,c. In addition, to accurately position sensors on the respective parts of the user’s body, the x-OSC [21] sensor with a battery is mounted in a smartphone sport belt, as shown in Figure 10c. Therefore, the inertial sensor data and the skeletons analyzed using the depth sensor were recorded for evaluation. The sensor data from 10 behaviors performed by 10 users were recorded; the representative snapshots of the behaviors are shown in Figure 11. In Figure 11, BP and BB are on-field behaviors of a baseball player; LS, RS, DS, LL, and RL are off-field behaviors during the warm-up of a baseball player; and NW, AW, and PC are off-field behaviors in a baseball player’s daily life. Moreover, each behavior of a user is repeated for 10 times in different trails. Therefore, a dataset with 10×10×10=1000 behavior trails was generated in the experiments.

### 4.1. Quantitative Evaluation

In the quantitative evaluation, to adopt the long short-term memory (LSTM) [19] network for training the behavior classifiers with deep neural networks, Tensorflow [22] libraries are used for implementing the proposed method. In addition, support vector machine (SVM) [23] is a widely used method to train classifiers for human behavior recognition from a depth camera sensor [24] and inertial sensors on a smart phone [25]. Therefore, SVM is implemented based on a built-in Matlab function [26] to train classifiers to compare behavior recognition capabilities with the proposed LSTM-based approach.

**Figure 12:**
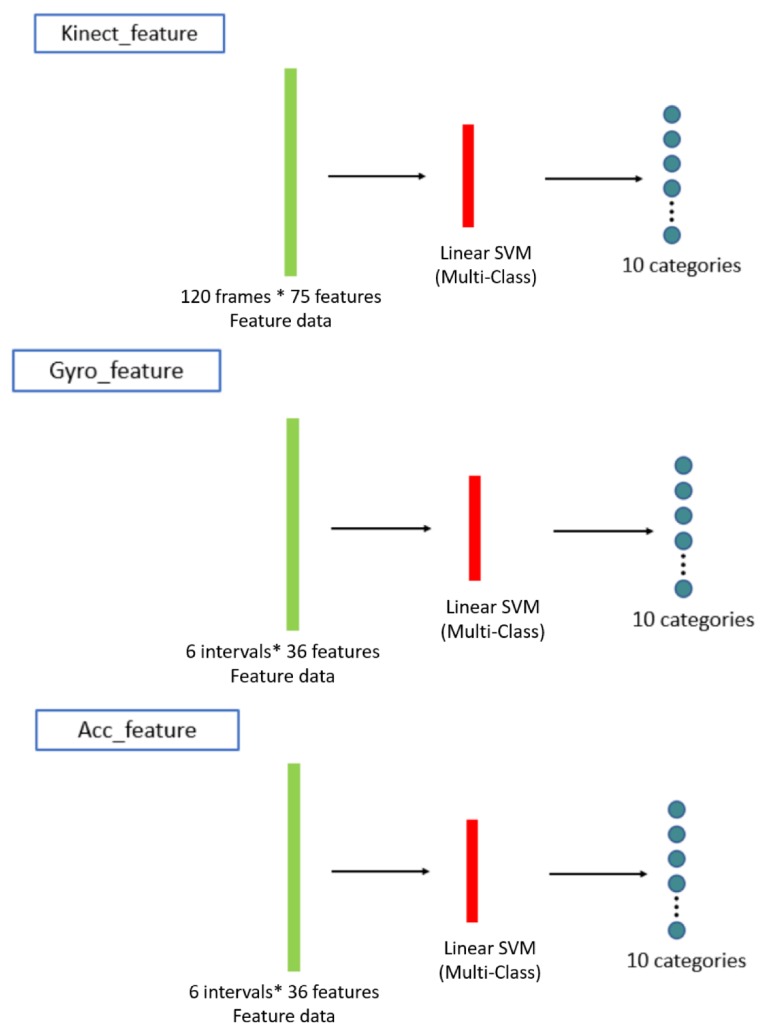
The training process for the behavior classifiers for a SVM-based scheme for performance comparison.

The confusion matrix in the following results are with the leave-p-out cross-validation [27] (p = 30 in our experiments). In the 10×10×10=1000 behavior trails, 30% of the trails are used for testing, and the other 70% of the trails are used for training the LSTM and the SVM models. To compare the performance of the proposed LSTM-based approach to a conventional SVM-based [23] approach in the experimental results, similarly, the 1D features are also taken as the input for training the behavior classifiers, as shown by the green rectangles in Figure 12. Furthermore, the SVM-based classifiers can be obtained for performance comparison.

**Figure 13:**
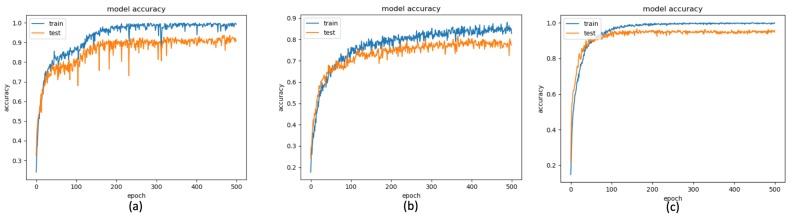
LSTM Accuracy of the depth-sensor only result (**a**) depth camera, (**b**) gyro sensor, and (**c**) accelerometer.

**Figure 14:**
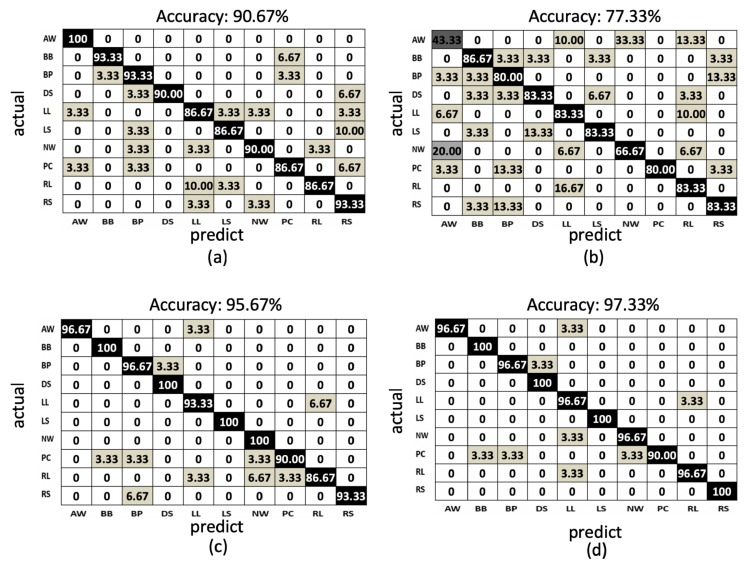
The confusion matrix of the LSTM-based results: (**a**) depth sensor, (**b**) gyro sensor, (**c**) accelerometer, and (**d**) decision fusion results.

**Figure 15:**
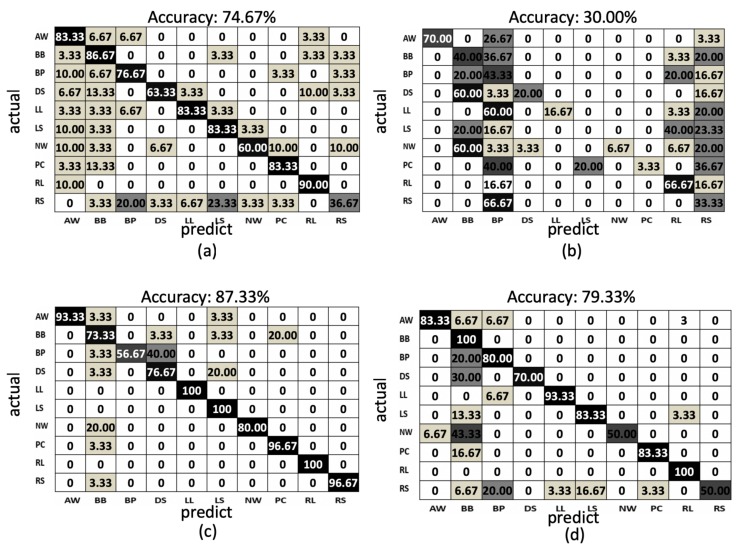
The confusion matrix of the SVM-based results: (**a**) depth sensor, (**b**) gyro sensor, (**c**) accelerometer, and (**d**) decision fusion results.

In the proposed method, using the LSTM network to train the behavior models from the proposed features, the model accuracies of the training phases and testing phases of the depth sensor only, gyro sensor only, and accelerometer only situations are depicted by the blue curves and orange curves in Figure 13a–c, among the observations from epoch 1 to epochs 500. The accuracy (orange curve) converges to 0.90, 0.77, and 0.95 at 200, 300, and 100 epochs for the depth sensor only, gyro sensor only, and accelerometer only modalities, correspondingly. To more completely obtain the training models from the three modalities, we use the classifier models obtained at the 500 epochs. Furthermore, the confusion matrices of the depth sensor only, gyro sensor only, and accelerometer only situations are depicted in Figure 14a–c, with the average accuracy of 90.67%, 77.33%, and 95.67%, respectively. Moreover, the average accuracy of the decision fusion is improved to 97.33%, and the confusion matrix is depicted in Figure 14d. Therefore, in the proposed LSTM-based behavior recognition scheme, with a decision fusion, the accuracy can be further improved. On the other hand, when the machine learning classifier is replaced by SVM, the average behavior recognition accuracy ranges from 30.00% to 87.33%, and the confusion matrices are shown in Figure 15a–c. However, because the average accuracy of the gyro sensor modality is low (30.00%), with a decision fusion process, as shown in Figure 15d, the fused average accuracy is only 79.33% with SVM-based classifiers.

Summarizing the average accuracy of the proposed LSTM-based approach and an SVM-based approach (Figure 14 and Figure 15) with different sensing modalities, Table 1 provides comparison results. The LSTM-based approach demonstrates substantially better average accuracy. The sequential relationships of the sensing data from multiple modalities can be retrieved using the proposed LSTM-based approach with deep neural networks. Although the gyro sensor modality is noisy in the behaviors to cause lower average accuracy, with a decision fusion process, the proposed LSTM-based approach still provides more accuracy than single modalities do to compensate for noisy situations occurring in the gyro sensor modality.

### 4.2. Qualitative Evaluation

**Figure 16:**
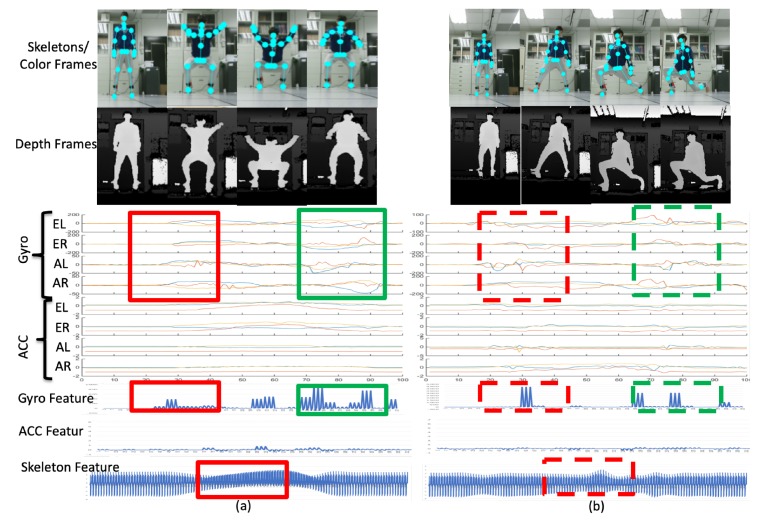
Representations of (**a**) the deep squat motion and (**b**) the left lunge motion.

Figure 16a depicts the deep squat (DS) motion and Figure 16b depicts the left lunge (LL) motion. The sensor signal distribution bounded by the red rectangles on the left in Figure 16a is quite different to the signal distribution bounded by the red dashed rectangles on the left in Figure 16b, which are observed in the corresponding time region. The distributions of the signals bounded by the green rectangles and the green dashed rectangles are quite different. Therefore, the DS and LL motions can be distinguished as different behaviors in the multiple modalities according to the proposed feature extraction process.

**Figure 17:**
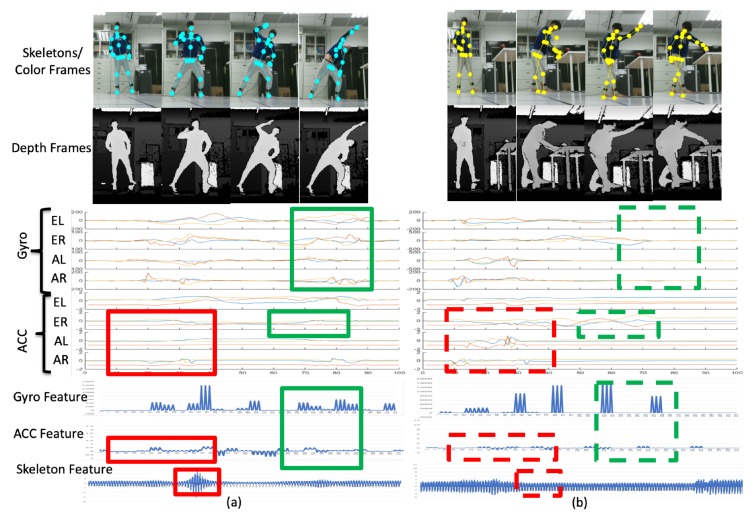
Representations of (**a**) the left-stretch motion and (**b**) the coffee-pouring motion.

Similarly, the left stretch (LS) motion (Figure 17a) and CP (Figure 17b) can be classified as two separate behaviors. For instance, during the period bounded by the red rectangle in Figure 17a, the accelerometer has stable distribution for the sensor ER, AR, and AL, because the feet and one of the user’s hands is not moving. Simultaneously, for the PC motion, the ER, AR, and AL sensors have large responses as per the signals bounded by the red dashed rectangle in Figure 17b, because the user’s hand and body are rotated and move the desk of the coffee machine. Furthermore, the user’s right hand is moved with a rotating motion to cause the ER sensor response indicated by the green dashed rectangle in Figure 17b. Consequently, the motion LS and PC can be distinguished from the signal distribution of the multiple modalities.

**Figure 18:**
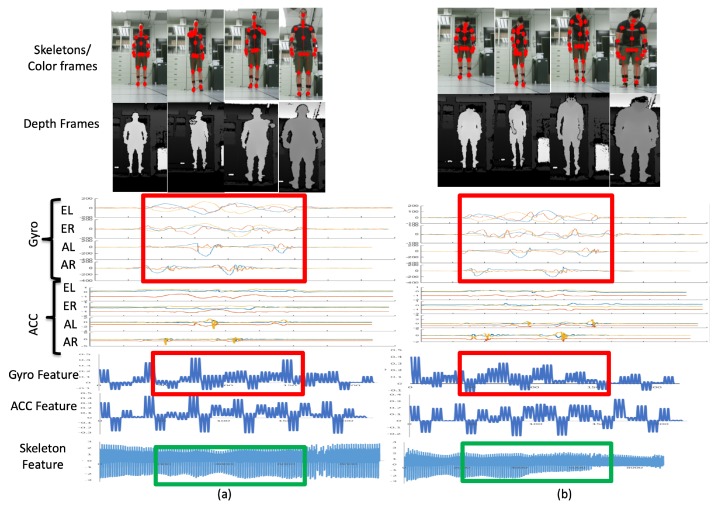
Results from AW to NW; similar gyro features, dissimilar skeletal features.

When two behaviors are very similar (Figure 18), abnormal walking (AW) behavior may be incorrectly identified as normal walking (NW) behavior in one of the modalities. The gyrosensor signal distributions (bounded by the red rectangle) of AW and NW are similar, and the resulting gyro features also share similar patterns. Therefore, according to the values in Figure 14b, 30% of AW is erroneously recognized as NW and 20% of NW is incorrectly recognized as AW. However, AW and NW behaviors in the camera modality have different distributions. As per the signals bounded by the green rectangles at the bottom of Figure 18, the skeleton feature obtained can distinguish two separate behaviors. The values in Figure 14c demonstrate that 0% of AW is erroneously recognized as NW and vice versa. As a result, even if behaviors may be erroneously recognized in one modality (gyro sensors in this case), the other modalities (depth cameras in this case) can compensate after a decision fusion process. As per the values in Figure 14d, 0% of AW is incorrectly recognized as NW and vice versa.

### 4.3. Complexity Comparison

The proposed deep-learning method is compared for an LSTM approach and an SVM approach. To offer mobile capability to collect different behaviors for on- and off-field activities, both approaches are operated on a laptop computer in a Windows 10 environment, with an Intel Core i7, a 2.70-GHz CPU, and 8 GB of RAM. The total computational time for operating the whole dataset is presented in Table 2. A total of 1000 sensing temporally synchronized data were collected for each trail. With a leave-30-out cross-validation, 300 trails were used for testing, and 700 trails were used for training. The total training time for the depth modality, gyro sensor modality, and accelerometer modality are included in the first to third rows of Table 2. The LSTM approach clearly requires more time than the SVM approach does to generate the deep-learning model. In addition, because the depth modality has many more features than the gyro sensor modality and the accelerometer modality do, the operational time for the depth sensor is much greater than for the other two modalities. However, when the behavior model is already obtained, in the testing phase, the operational time for the proposed decision-level fusion behavior recognition method of the LSTM and the SVM approaches is in the same time scale. Comparing Table 1 and Table 2, we may conclude that the proposed LSTM-machine learning modeling achieves higher behavior recognition accuracy with spending much more computational time in the training phase. By contrast, in the testing phase, more computational time is needed for the LSTM approach as for the SVM approach.

## 5. Conclusions

The proposed method can recognize on- and off-field behaviors of baseball players based on the obtained LSTM classifiers trained from multimodal IoTsensors. A novel baseball player behavior classification system is proposed. In the past, researchers considered only on-field activities; however, both on- and off-field activities provide essential information for evaluating players’ performance and status. Among the 10 behaviors proposed, baseball pitch and baseball bat are used to identify players’ on-field performance; left and right stretch, left and right lunge, and deep squat are used to understand players’ status during warm-up or during workouts; normal or AW and CP behaviors are used for daily general behavior classification. The contribution of this paper is threefold: (i) the data from a depth camera and multiple inertial sensors can be segmented with a fusion manner, (ii) the skeletal positions with time-variant skeleton vector projection and the statistical properties are extracted as the features, and (iii) a decision-level fusion with a deep-learning process is proposed to train the behavior classifiers. The preliminary results of the proposed baseball player behavior classification system demonstrate that the on- and off-field behaviors of a potential baseball player can be analyzed by multimodal sensing data for further evaluation by a baseball coach. In the future, a player’s body status, not only the physical positions but also the internal muscle status can be analyzed by deep-learning technologies using big data collected from baseball players.

## Figures and Tables

**Table 1:** Comparison results of average accuracy.

	LSTM	SVM
Depth Sensor	90.67%	74.67%
Gyro Sensor	77.33%	30.00%
Accelerometer	95.67%	87.33%
Decision Fusion	97.33%	79.33%

**Table 2:** Comparison results of computational time (s).

	LSTM	SVM
Depth Sensor Training Time	1637.92	2.92
Gyro Sensor Training Time	135.64	0.32
Accelerometer Training Time	134.61	0.48
Decision Fusion Testing Time	0.49	0.32
